# Intravascular papillary hemangioendothelioma disguised as a peripheral sheath tumor of median nerve at the wrist: a case report and literature review

**DOI:** 10.1007/s00256-022-04250-y

**Published:** 2022-12-14

**Authors:** Vrajesh J. Shah, Kerry Sung, Vanessa Goodwill, Brady K. Huang, Reid Abrams

**Affiliations:** 1 School of Medicine, University of California San Diego, San Diego, CA USA; 2 School of Medicine, Rush University, Chicago, IL USA; 3 Department of Pathology, University of California San Diego, San Diego, CA USA; 4 Department of Radiology, Division of Musculoskeletal Imaging, University of California San Diego, San Diego, CA USA; 5 Department of Orthopedic Surgery, University of California San Diego, San Diego, CA USA

**Keywords:** Intravascular papillary hemangioendothelioma, Masson’s vegetant, Carpal tunnel, Peripheral nerve sheath tumor, MRI

## Abstract

**Background:**

Intravascular papillary hemangioendothelioma (IVPH) is a benign lesion previously reported in the nasal cavity, neck, upper extremities, and breast. Diagnosis with cross-sectional imaging can prove difficult, with histopathological examination necessary for diagnosis. IVPH resulting in carpal tunnel symptoms is quite rare.

**Case presentation:**

We report the case of a 37-year-old woman who presented with a radial, volar right wrist mass enlarging over the span of 5 years. She noted numbness and tingling in her wrist and thumb, exacerbated by minor accidental collisions and wrist hyperextension. There was no antecedent trauma. On examination, a mildly tender, mobile mass was evident at the volar aspect of the right wrist. Magnetic resonance imaging (MRI) with contrast demonstrated a lobulated, predominantly T2 hyperintense, heterogeneously enhancing mass thought to be a peripheral nerve sheath tumor. The patient elected for surgical excision of the mass, and the histopathological examination showed organizing thrombi with prominent papillary endothelial hyperplasia. At the 2-month follow-up, the patient had full range of motion of her fingers and wrist, with subjectively normal sensation in the distribution of the median nerve.

**Conclusion:**

Carpal tunnel syndrome, in exceedingly rare occasions, can result from an IVPH. MRI findings may be confused with more common entities. Histopathological confirmation remains necessary for conclusive diagnosis.

## Introduction

Since its original description as a hemorrhoidal vein tumor, intravascular papillary hemangioendothelioma (IVPH) has been established as a benign lesion. It has been reported to be found in the nasal cavity, breast, neck, and hand [1–5]. A rare extravascular manifestation of IVPH can be found in the central nervous system, where it can cause mass effect, increased intracranial pressure, compress adjacent tissues, and cause spinal cord compression [2, 6–9]. Previously, a single case of IVPH resulting in carpal tunnel syndrome had been reported, but lacked radiologic-pathologic correlation [10].

IVPH can be challenging to diagnose both clinically and with advanced cross-sectional imaging. Histopathological analysis is necessary for a conclusive diagnosis. IVPH of the peripheral nervous system (PNS) can present with a variety of clinical syndromes depending on the affected peripheral nerve. It is known to share common imaging features with neurogenic tumors and tenosynovial giant cell tumors, with varying magnetic resonance imaging (MRI) characteristics for primary and secondary IVPH [11]. The pathologic diagnosis of IVPH can be equally elusive. It is also referred to as a pseudoangiosarcoma, with features mimicking angiosarcomas. We present a rare case, complete with radiologic-pathologic correlation, where a carpal tunnel release was performed for an IVPH, preoperatively diagnosed as a peripheral nerve sheath tumor of the median nerve.

## Case

The patient is a 37-year-old right-hand dominant woman who first presented at an outside facility for examination of a radial, volar right wrist mass. She initially noted the hard mass in 2016. She experienced nerve-like pain, numbness, and tingling from her wrist to thumb with exercise. She denied any prior trauma. Electromyography did not reveal nerve damage. Magnetic resonance imaging (MRI) suggested a peripheral nerve sheath tumor. At the time, the patient declined surgical intervention as her symptoms did not greatly impede her daily life. She presented to us 3 years later, reporting interval growth of the mass and pain with hyperextension loading and minor accidental collisions.

Focused examination of her right hand and wrist revealed a 3.0 × 2.0-cm mobile mass at the volar aspect of the right wrist, just beneath the palmaris longus tendon in the distal forearm, which was mildly tender to palpation. No subjective sensory deficits were noted to light touch. The patient had full strength, and no thenar atrophy was appreciated. There was a negative Tinel sign over the mass.

**Fig. 1:**
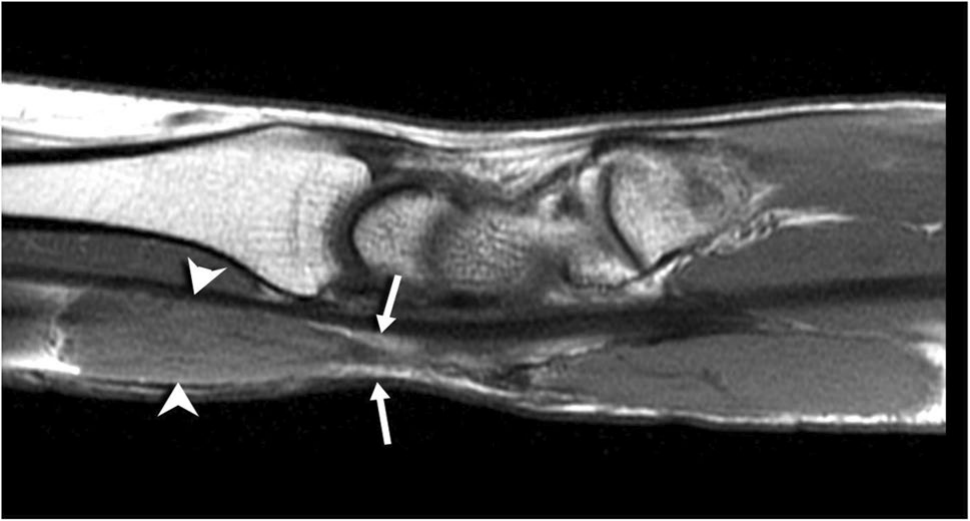
Sagittal T1-weighted fat-suppressed MR image shows the presence of a “split fat” sign and “tail sign,” with hyperintense adipose tissue (fat) surrounding the exiting median nerve (arrows) at the distal end of the mass. The mass itself is isointense to skeletal muscle (arrowheads)

**Fig. 2:**
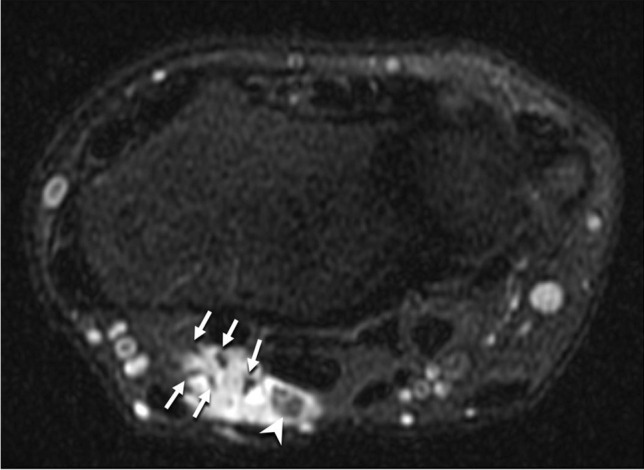
Axial T2-weighted fat-suppressed MR image just proximal to the radiocarpal joint shows multiple hypointense nerve fascicles of the median nerve coursing through a predominately hyperintense mass. Scattered regions of hypointensity (arrowhead) were present within the mass, likely reflecting localized areas of thrombosis

**Fig. 3:**
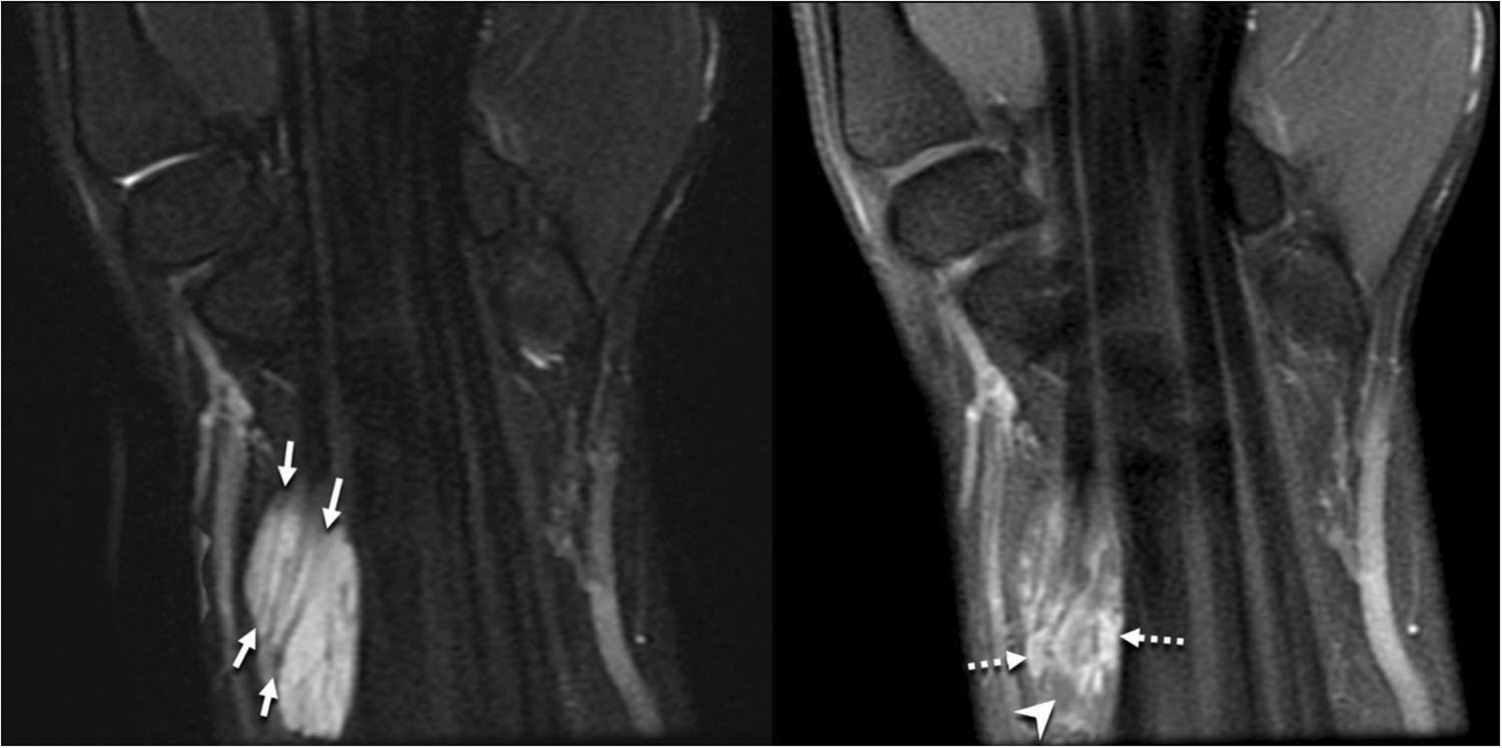
Coronal T2-weighted fat-suppressed MR image (left) shows multiple hypointense nerve fascicles (arrows) of the median nerve coursing through a predominately hyperintense mass. Coronal T1-weighted fat-suppressed MR image (right) following the intravenous administration of 4.5 mL of a gadolinium-based contrast agent shows nodular areas of enhancement within the mass (dashed arrows), as well as scattered non-enhancing areas (arrowhead)

Multiplanar multisequence MRI of the right wrist was obtained before and after 4.5 mL of an intravenous gadolinium-based contrast. This showed a lobulated, predominantly T2 hyperintense, heterogeneously enhancing mass measuring 2.0 cm transverse × 1.2 cm dorsal-volar × 3.1 cm in length along the volar-radial aspect of the distal forearm/wrist. T1-weighted sequences demonstrated the “split fat” and “tail sign” indicating the neural origin of the mass (Fig. 1). T2-weighted sequences revealed small, rounded areas of intermediate to low signal with the mass noted (Fig. 2). The mass was located along the course of the median nerve, deep to the palmaris longus and flexor carpi radialis tendons with nodular areas of internal enhancement (Fig. 3) and was reported to be consistent with a peripheral nerve sheath tumor.

**Fig. 4:**
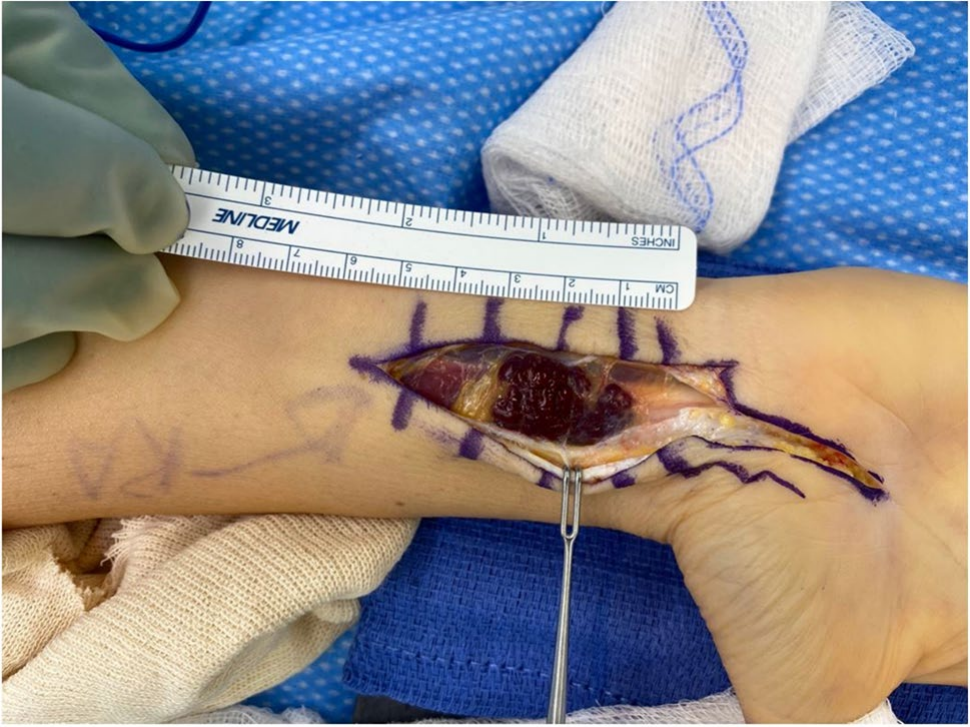
Intraoperative photograph reveals gross appearance of the superficial mass after superficial dissection of overlying dermis. A roughly 3.0 cm × 2.0 cm vascular “bag of worms” fungating mass was appreciated, tracking along the median nerve. The palmaris longus tenon is radially retracted

**Fig. 5 A:**
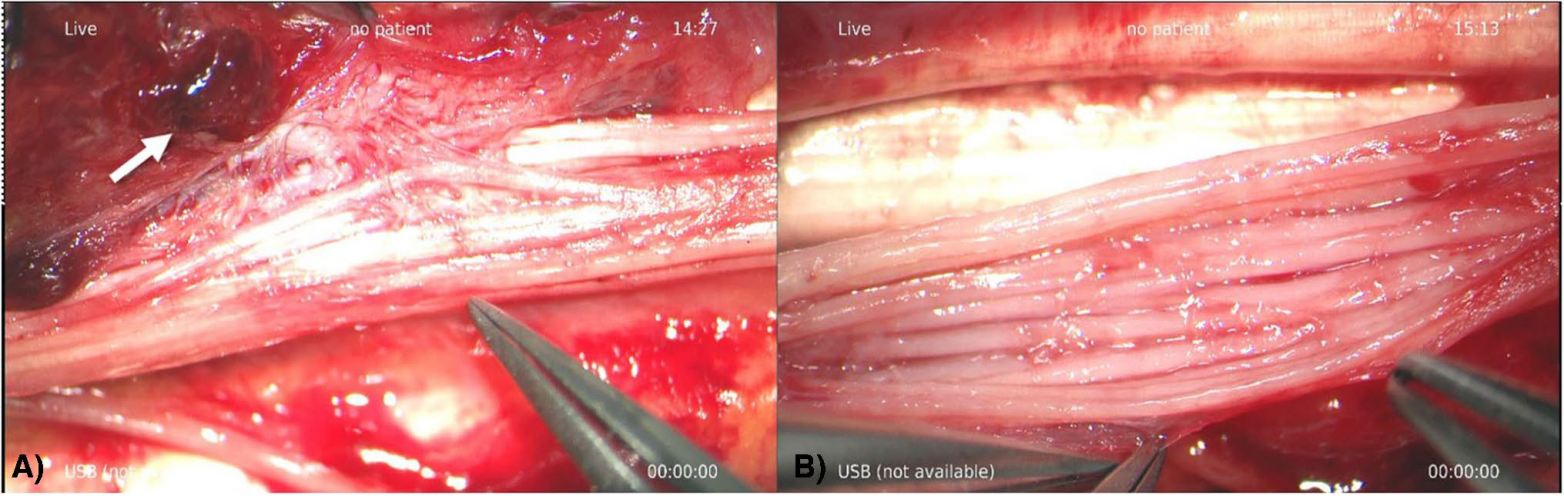
Intraoperative image taken under microscopic magnification. The interdigitation of the vascular mass in between the fascicles of the median nerve is evident. Note the areas of thrombus peripherally (white arrow). **B** Intraoperative photograph under microscopic magnification taken after the IVPH was meticulously dissected from the fascicles of the median nerve. The nerve remained intact and the patient denied any neurological deficits in the affected extremity during the 2-month post-operative visit

The patient elected to undergo elective surgical removal of the symptomatic, enlarging mass. On the day of surgery, she was induced under general anesthesia, prepped, and draped in a typical sterile fashion. An extensive carpal tunnel approach was drawn, and an incision was made in the distal forearm ulnar to palmaris longus. After careful dissection of the superficial soft tissue to the volar forearm fascia with loupe magnification, the palmaris longus was radially retracted to expose the median nerve. A large, fragile, seemingly vascular mass was examined with the median nerve (Fig. 4). Under microscopic magnification, a meticulous dissection of the fragile mass from the fascicles of the median nerve was performed. The tumor appeared to have a “bag of worms” network of infiltrative vascular channels interdigitated among the fascicles of the median nerves (Fig. 5). A carpal tunnel release was performed to prevent nerve compression from the inflammatory response secondary to extensive fascicular manipulation. The wound was then copiously irrigated and reapproximated.

**Fig. 6 A:**
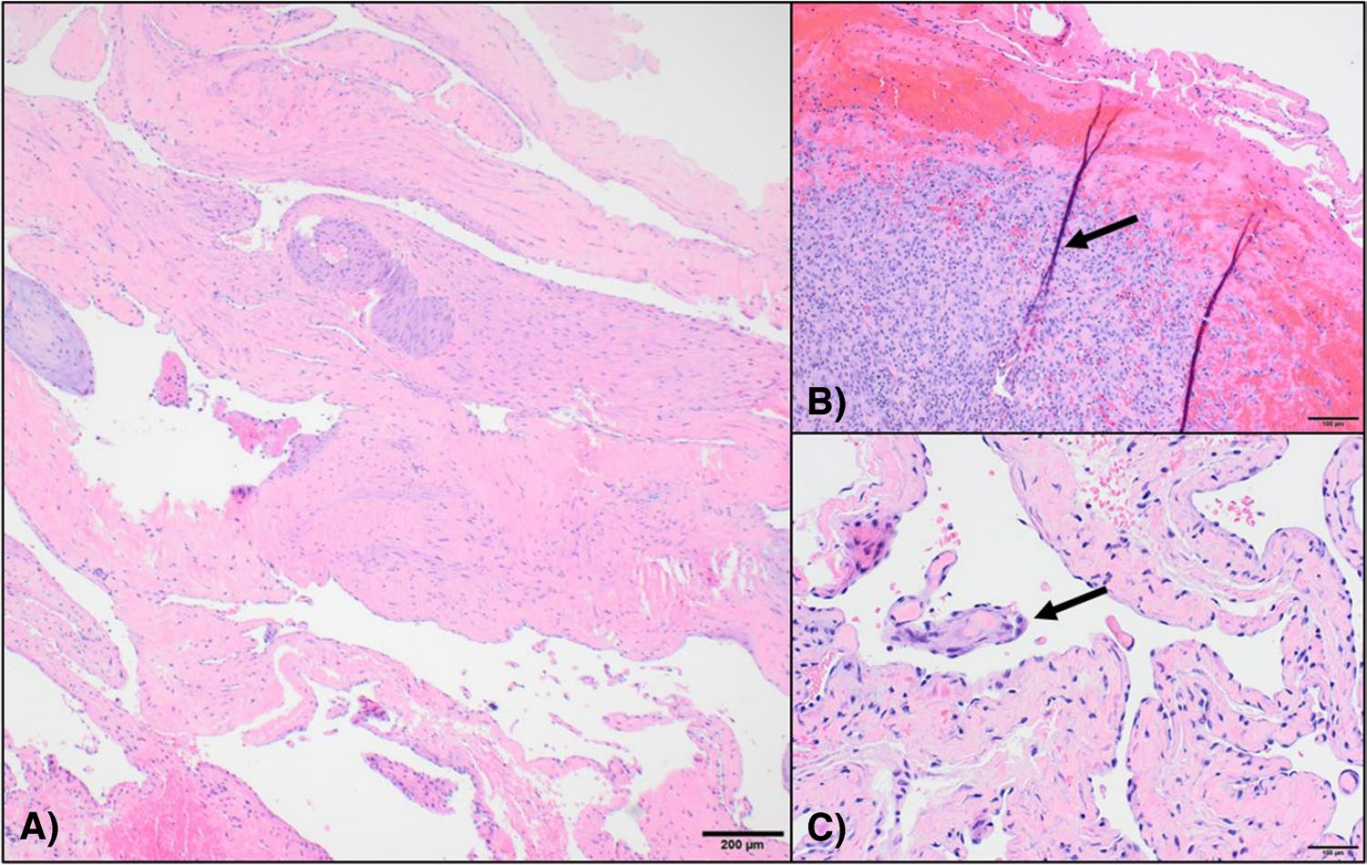
At 4× magnification, hematoxylin and eosin (H&E) stain of the resection specimen showed multiple irregularly contoured vascular channels lined by endothelial cells and separated by dense fibrous connective tissue. **B** At 10× magnification, the image shows organizing thrombus with active granulation tissue (black arrow). C) At 10× magnification, the image shows multifocal endothelial covered papillae (black arrow) with central collagenous cores throughout vascular lumens, consistent with papillary endothelial hyperplasia (aka IVPH)

Histopathology of the specimen revealed multiple irregularly contoured vascular caverns lined by endothelial cells with dense fibrous connective tissue septae, features suggestive of a cavernous hemangioma (Fig. 6). Additionally noted within the vascular spaces were multiple small organizing thrombi and focally prominent papillary endothelial hyperplasia typical of IVPH.

The patient recovered well after her right median nerve release and extensive internal neurolysis with removal of the IVPH. At her 2-month follow-up, her wound was healing well and non-tender to touch. She had full range of motion of her fingers and wrist, and sensation in the median nerve distribution was subjectively normal.

## Discussion

Historically, IVPH was commonly referred to as Masson’s vegetant hemangioendothelioma. First described by Masson to be neoplastic, it was later determined to be a benign composition of reactive thrombi encompassed by a proliferative papillary endothelium [4]. IVPH accounts for less than 2% of soft tissue masses, occurring in women more than men [12]. There are three current classifications for IVPH: pure, mixed, and secondary. Hashimoto studied 91 cases of intravascular papillary endothelial hyperplasia and stated 33% are of the pure form, while 60% have mixed characteristics [13]. The study describes the pure form to be a less than 2-cm mass in middle-aged women arising from dilated vascular spaces without a pre-existing lesion. While more common, the mixed form is found in younger patients roughly 28 years of age, are intramuscular, greater than 2 cm, and occur from pre-existing vascular lesions [4]. Based on our pathological findings, our patient likely had the pure form of IVPH.

There are several theories to the pathogenesis of this variant hemangioma. A prior study suggests the etiology may be hormonal, given its resemblance to other benign vascular masses [14]. Another theory defines the role of fibroblast growth factor beta due to its ability to induce thrombus formation and endothelial proliferation [15]. Others believe trauma to play a major factor, which is less likely contributory to this case, as our patient did not report any such history [16].

Although the composition of the tumor is benign in nature, IVPH must be differentiated from other soft tissue tumors. The differential diagnosis includes venous malformations, angiovenous malformations, pyogenic granulomas, schwannomas, neurofibromas, and Dabska tumors. In addition to the aforementioned differential considerations, key diagnoses to consider are angiosarcomas and Kaposi sarcomas, as both are malignant and aggressive in nature [17, 18]. It is important that these diagnoses are considered, as mixed IVPH have features that mimic these conditions.

MRI imaging characteristics are known to correlate histopathologically to help narrow the differential and offer a preliminary diagnosis. Venous malformations can be characterized as lobulated masses with intermediate to low signal on T1-weighted images, with increased intensity on T2-weighted images [19]. Angiovenous malformations have features of tangled signal voids with feeding vasculature and tissue infiltration on both T2- and T1-weighted images [19]. Pyogenic granulomas have been described to be iso-intense to muscle on T1-weighted and to veins on T2-weighted images [19]. Schwannomas can have a characteristic target or split fat sign, and have a general appearance of being iso-or hypointense on T1 with mixed enhancement [20]. A neurofibroma has similar characteristics to schwannomas on T1-weighted images and can exhibit a target-sign on T2-weighted images with a central low signal intensity region and a T2-hyperintense rim [21]. Ancient schwannomas can also have highly variable appearance compared to conventional schwannomas on both T1- and T2-weighted images due to varying distributions of Antoni A and Antoni B areas and hemorrhage, cystic degeneration, and necrosis [22]. Dabska tumors are hypointense on T1-weighted images with a hyperintense lobular pattern on T2-weighted images [23]. Angiosarcomas show evidence of aggressive, infiltration of surrounding tissues with intermediate T1 signal and high T2 signal [19].

For a definitive diagnosis, histopathological examination is required. Histologic features characteristic to IVPH are papillary-like proliferation of the endothelium, proximity to a dilated vessel wall, lack of infiltration of adjacent soft tissue, and organized intraluminal thrombi formation [13]. In retrospect, the imaging features of this case may have suggested the diagnosis, as evidenced by nodular enhancement within the lesion and hypointense areas that likely represented organizing thrombus. Once confirmed on histopathology, treatment offers a good prognosis. Surgical excision results in an excellent prognosis, with a few reported cases of recurrence at which radiation therapy may be considered [24].

We report a rare case with radiologic-pathologic correlation, in which a carpal tunnel release and internal neurolysis performed for a soft tissue mass initially thought to be a peripheral nerve sheath tumor. Surgical resection and subsequent histopathological examination revealed intravascular papillary endothelial hyperplasia and findings consistent with IVPH.
